# Role of Oxidative DNA Damage and Repair in Atrial Fibrillation and Ischemic Heart Disease

**DOI:** 10.3390/ijms22083838

**Published:** 2021-04-07

**Authors:** Liangyu Hu, Zhengkun Wang, Claudia Carmone, Jaap Keijer, Deli Zhang

**Affiliations:** Human and Animal Physiology, Wageningen University & Research, 6708 WD Wageningen, The Netherlands; liangyu.hu@wur.nl (L.H.); zachwang0317@hotmail.com (Z.W.); claudia.carmone@wur.nl (C.C.); jaap.keijer@wur.nl (J.K.)

**Keywords:** oxidative DNA damage, DNA repair, cardiac disease, atrial fibrillation, ischemic heart disease, ischemia/reperfusion injury, antioxidant, PARP1, NAD^+^, vitamin B3

## Abstract

Atrial fibrillation (AF) and ischemic heart disease (IHD) represent the two most common clinical cardiac diseases, characterized by angina, arrhythmia, myocardial damage, and cardiac dysfunction, significantly contributing to cardiovascular morbidity and mortality and posing a heavy socio-economic burden on society worldwide. Current treatments of these two diseases are mainly symptomatic and lack efficacy. There is thus an urgent need to develop novel therapies based on the underlying pathophysiological mechanisms. Emerging evidence indicates that oxidative DNA damage might be a major underlying mechanism that promotes a variety of cardiac diseases, including AF and IHD. Antioxidants, nicotinamide adenine dinucleotide (NAD^+^) boosters, and enzymes involved in oxidative DNA repair processes have been shown to attenuate oxidative damage to DNA, making them potential therapeutic targets for AF and IHD. In this review, we first summarize the main molecular mechanisms responsible for oxidative DNA damage and repair both in nuclei and mitochondria, then describe the effects of oxidative DNA damage on the development of AF and IHD, and finally discuss potential targets for oxidative DNA repair-based therapeutic approaches for these two cardiac diseases.

## 1. Introduction

Cardiac diseases, a class of disorders affecting biological structure and/or physiological function of the heart, are the leading cause of morbidity and mortality worldwide. In particular, atrial fibrillation (AF) and ischemic heart disease (IHD) emerge as the most common and serious cardiac diseases in clinical practice [1,2]. AF, the most common heart rhythm disorder, is characterized by the rapid and irregular beating of the upper atrial chambers due to the electrical, structural, and functional remodeling of atrial cardiomyocytes [3,4]. This arrhythmia can result in static atrial blood, promoting the formation of atrial thrombi and triggering detrimental symptoms, such as stroke, arterial embolization, and a reduced quality of life [5,6,7]. AF is associated with an increased risk of death in patients with IHD [8]. Those two cardiac disorders share some similar symptoms, such as angina, arrhythmia, heart muscle damage, and loss of cardiac muscle activity [9]. Specifically, the structural changes of atrial cardiomyocytes after sustained AF closely resemble the changes in ventricular myocytes due to chronic low flow ischemia [10,11]. IHD is caused by an insufficient supply of oxygen due to the restriction of blood flow into the cardiac muscles, which occurs mainly as a result of blockage (e.g., atherosclerosis, thrombus, coronary artery stenosis) in the arteries of the heart [12]. One of the most typical consequences of IHD is ischemia/reperfusion injury (IRI). As early as 1960, Jennings et al. found that the reperfusion process accelerated the development of myocardial death in a canine model of IHD [13]. After more than half a century of experimental research and clinical practice, IRI has been proven to contribute to numerous cardiovascular diseases. Despite some exciting and innovative improvements in clinical management of AF and IHD, treatment modalities for two diseases still have limited efficacy and safety [14,15], and a better understanding of the molecular mechanisms promoting AF and IHD is needed to improve the treatment.

In the past decades, various mechanisms underlying development of AF and IHD have been identified. Apart from well-known environmental or genetic mutation-mediated risk factors associated with AF and IHD [16,17,18], recent evidence suggests that oxidative stress-induced DNA damage occurs and plays a key role in the pathophysiology of these two cardiac diseases [19,20,21]. Oxidative DNA damage is caused by oxidative stress, which is commonly characterized by abnormal accumulation of mitochondrial reactive oxygen species (ROS) and the insufficient ability to detoxify these free radicals [22,23]. To overcome oxidative stress-induced DNA damage, eukaryotes have developed a complex set of DNA repair pathways [24]. An increasing number of studies have revealed that reducing oxidative DNA damage by manipulating enzymes associated with oxidative DNA repair pathways or by supplementation of antioxidants and/or nicotinamide adenine dinucleotide (NAD^+^) could effectively inhibit cardiac damage associated with heart diseases, including AF and IHD [22,25,26,27]. Here, we summarize the mechanisms underlying ROS-induced oxidative DNA damage and its possible role in pathogenesis of AF and IHD. Furthermore, we discuss the potential therapeutic treatments targeting oxidative DNA damage and repair to delay the onset and progression of cardiac diseases.

## 2. Oxidative Stress and Oxidative DNA Damage

### 2.1. ROS and Oxidative Stress

Living cells are continuously exposed to potentially detrimental free radicals, which are derived intracellularly or extracellularly. Free radicals are defined as atoms or molecules with one or more unpaired electrons, enabling their highly reactive activity [28]. Among these free radicals, ROS are highly reactive and unstable molecules containing oxygen, which have been implicated in the pathogenesis of heart diseases [29]. Imbalanced ROS levels in AF leads to morphological and functional changes in the affected human cardiac myocytes, leading to an oxidative vicious cycle [30]. Reduction of mitochondrial ROS prevents and reverses electrical instability responsible for sudden cardiac death and chronic remodeling in heart failure [31].

ROS are produced by multiple biochemical reactions in several cellular systems localized on the plasma membrane, membranes of mitochondria and endoplasmic reticulum, and in the cytosol and peroxisomes [32,33]. The mitochondrial electron transport chain is considered as the predominant source of ROS precursors [34], while NADPH oxidases (NOXs) in the plasma membrane also represent one of the major endogenous sources of ROS [35,36,37]. Interestingly, there is a substantial interplay between these two sources: NOXs could increase mitochondrial ROS, which further activates the cytoplasmic NOXs and promotes cellular superoxide production [38,39]. In cardiomyocytes, NOX2 was reported to amplify mitochondrial ROS levels and inhibition of NOX2 attenuated mitochondrial dysfunction and decreased mitochondrial ROS [40]. Similarly, the NOX4 isoform is also a major source of mitochondrial oxidative stress in the failing heart [41], together indicating a crosstalk of NOXs and mitochondrial ROS in cardiac pathophysiological processes. 

Net ROS emission is determined not only by the ROS formation rate but also by ROS elimination via various antioxidant defense systems. Highly reactive and damaging superoxide is rapidly converted to hydrogen peroxide by superoxide dismutases (SODs). Hydrogen peroxide is further inactivated primarily by catalases in peroxisomes and cytoplasm [42] and by the glutathione, peroxiredoxin, and thioredoxin dependent systems in mitochondria, which are regenerated at the expense of NADPH [43,44,45]. The role of hydrogen peroxide in the human body is like a “double-edged sword”. At unharmful physiological levels, hydrogen peroxide is intrinsic for maintaining normal cellular functions and is involved in the regulation of metabolic processes as well as in immune response and cellular differentiation [46]. However, unconverted superoxide and high levels of hydrogen peroxide and other ROS lead to cell damage or death mainly by altering membrane and DNA integrity [47], which are characteristics of oxidative stress. 

Oxidative stress is caused by an imbalance between production of ROS and antioxidant defense system capacity, which can result in pathophysiological changes in the body, contributing to induction of cancers, metabolic syndromes, neurodegenerative diseases, inflammatory diseases, age-related diseases, and heart diseases [48,49]. Oxidative stress causes damage to major cellular macromolecules, including protein, lipid and DNA. Notably, oxidative DNA damage encompassed both nuclear DNA damage and mitochondrial DNA damage, which are both involved in the pathogenesis of various cardiac diseases, including AF and IHD.

### 2.2. Oxidative DNA Damage in Nuclei

**Figure 1:**
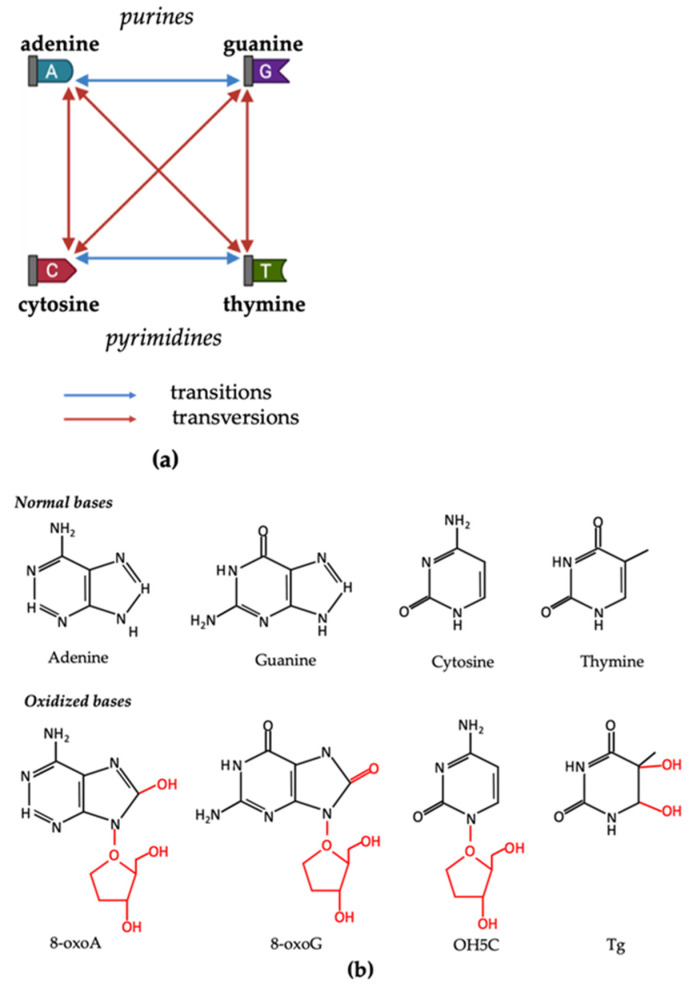
ROS-induced structural modifications on four DNA nucleobases—adenine, cytosine, guanine, and thymine. (**a**) DNA transitions and transversions. Blue lines represent transitions (changes between adenine and guanine, or cytosine and thymine). Red lines represent transversions (changes between purines and pyrimidines). (**b**) On top, nucleotides in DNA molecules: adenine, guanine, cytosine, thymine; and below, their major oxidized products: 8-oxo-2′-deoxyadenosine (8-oxoA), 8-oxo-2′-deoxyguanosine (8-oxoG), 5-hydroxy-2′-deoxycytidine (OH5C), and thymine glycol (Tg).

The oxidative DNA damage in nuclei can occur at multiple sites, such as nucleobases, nucleotides, and single- or double-strands of DNA molecules. Structural modifications of four DNA nucleobases—adenine, cytosine, guanine and thymine—represent the most common targets of oxidative damage caused by ROS [21]. When the structure is altered, base-pairing is subsequently disturbed, causing either DNA transition (changes between adenine and guanine, or cytosine and thymine) or transversion (changes between purines and pyrimidines) [50] (Figure 1a). Among the nucleobases, guanine is the most frequently oxidized base due to its low oxidation potential compared with other bases [51]. The most common oxidized form of guanine, 8-oxo-2′-deoxyguanosine (8-oxoG), is one of the most widely studied oxidative DNA lesions and is currently investigated as a biomarker for oxidative DNA damage [52,53]. Apart from guanine, the other three bases also have their major oxidized forms. The oxidation product of adenine, 8-oxo-2′-deoxyadenosine (8-oxoA), shares a similar structure with that of guanine. However, the yields of 8-oxoA are much lower than 8-oxoG, possibly due to the lower oxidation potential of adenine in comparison with guanine [54]. Free radicals usually attack thymine at multiple positions, generating various lesions. The most common oxidation product of thymine is thymine glycol, which is produced by the oxidation on the 5,6-double bond [55]. This bond is also the oxidative target of cytosine, whose main product is 5-hydroxy-2′-deoxycytidine [56] (Figure 1b). 

Oxidative base lesions in DNA leads to a considerable mutagenic potential via base misincorporation, mispairing, and substitution. Additionally, they also have non-mutagenic consequences, including the induction of a replicative block at the site of lesions, large deletions in DNA, and increased frequency of microsatellite instability [53,57]. In recent decades, ROS-induced DNA lesions have been proven to be vital in the pathogenesis of several cancers and cardiovascular diseases, making it a focus of human health. In atrial cardiomyocytes, DNA damage was associated with electrophysiological deterioration, including reduction in cardiomyocyte excitability and increase in dispersion of action potential duration, thereby creating a molecular and structural substrate for further arrhythmia [26]. Increased oxidative damage to DNA can also promote inflammation by increasing cytokine production, which causes cardiac structural and electrical remodeling [58]. Martinet et al. showed that oxidative DNA damage and inflammation were significantly increased in human atherosclerotic plaques [59], which are common cause of IHD. 

Taken together, excessive ROS can cause DNA damage in nuclei, with 8-oxoG as the most widely occurring oxidative DNA lesion. Oxidative DNA damage is involved in the pathogenesis of the cardiovascular diseases, including AF and IHD.

### 2.3. Oxidative DNA Damage in Mitochondria

Apart from the oxidative DNA damage in the nuclei, oxidative DNA damage also occurs in mitochondria. In fact, mitochondria are the predominant source of ROS in cells because of the high electron flux in electron transport complexes as part of oxidative phosphorylation (OXPHOS) to generate ATP. Mitochondria are also the only organelle, besides nuclei, containing their own DNA and machinery for synthesizing RNA and proteins [60]. All 13 mitochondrial DNA (mtDNA) encoded proteins are essential components of OXPHOS complexes I, III, IV, and V [61], and mutations in mtDNA can directly impact the essential function of ATP production and the concomitant ROS generation. MtDNA does not contain histones, and hence is thought to be more prone to oxidative damage [62]. Moreover, it is commonly believed that mtDNA is located near the mitochondrial inner membrane and the electron transport system, enhancing its susceptibility to ROS damage [28,63]. Indeed, mitochondrial ROS overproduction was associated with a high mutation rate of the mitochondrial genome [64], and ROS were reported to induce a rapid increase of mtDNA damage [65,66]. Mitochondria also possess several pathways for the repair of mtDNA damage, in particular the base excision repair (BER) pathway [67,68], which is described in detail below. Deficient repair of damaged mtDNA can lead to a dramatic accumulation of mtDNA molecules harboring deletions and a significant reduction in mtDNA copy number [61,69], which was shown to be associated with a higher risk of cardiovascular diseases, including sudden cardiac death [70]. Furthermore, various studies show that continuous damage to mtDNA can eventually result in nuclear mutations of genes encoding mitochondrial proteins, further triggering mitochondrial dysfunction in various diseases, suggesting a crosstalk between mtDNA and nuclear DNA [71,72,73,74].

Notably, mtDNA damage is widely involved in cardiac pathophysiology. Indeed, mtDNA damage in association with mitochondrial dysfunction appears to play a role in heart failure, both in humans and in animal models [75]. Ischemic hearts also display increased mtDNA damage and disturbed OXPHOS gene expression [76]. A recent study revealed circulating mitochondrial DNA, previously used as the biomarker for mitochondrial dysfunction and stress [77], as a possible biomarker for AF progression [78]. In brief, mtDNA in circulation could be released from cardiomyocytes in the blood upon stress, and the circulating levels were remarkably increased depending on different stages of AF and gender. Therefore, this circulating mitochondrial DNA has the potential to be applied in risk stratification of AF patients in a gender-specific manner.

Taken together, mtDNA is more susceptible to ROS damage than nuclear DNA. Moreover, mtDNA damage and repair were shown to play a key role in the pathogenesis of cardiac diseases.

## 3. Oxidative DNA Repair Pathways

To protect DNA molecules from oxidative damage, cells harbor a number of well-developed DNA repair processes. BER and nucleotide excision repair (NER) are two of the most important DNA repairing pathways. Additionally, increasing evidence shows that a minor process, DNA mismatch repair (MMR), might also play a considerable role in repairing oxidative DNA damage. All these pathways are thus potential therapeutic targets for cardiac diseases associated with oxidative DNA damage, such as AF and IHD.

### 3.1. Base Excision Repair (BER)

**Figure 2:**
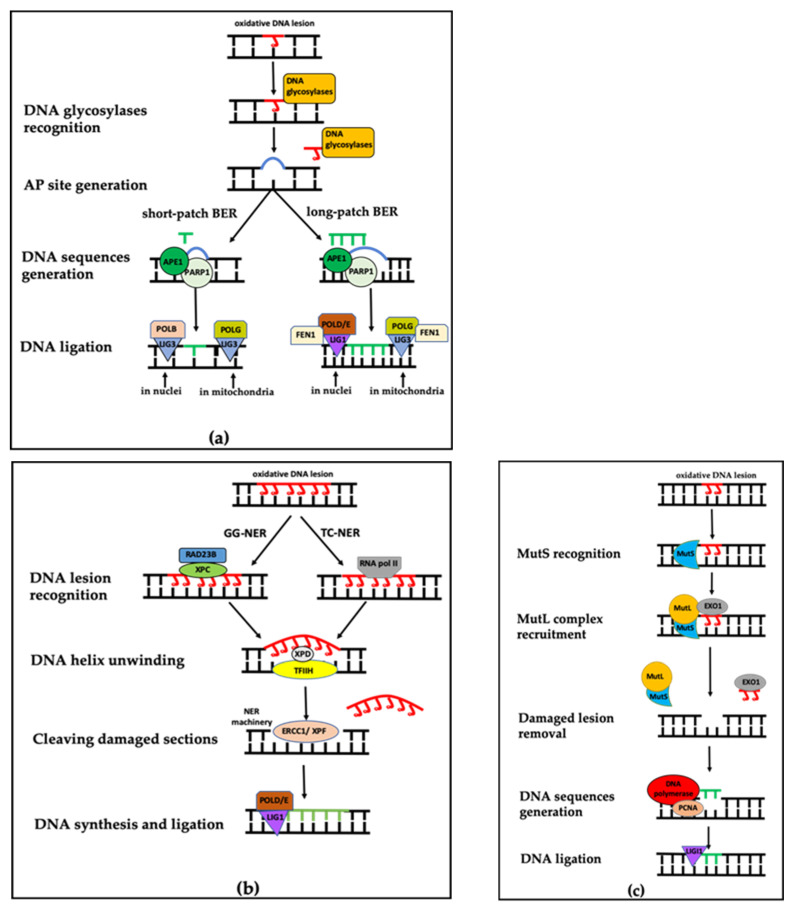
Overview of base excision repair, nucleotide excision repair and mismatch repair pathway. (**a**) Base excision repair (BER) pathway, including short-patch and long-patch BER sub-pathways. APE1: apurinic/apyrimidinic (AP) endonuclease 1; PAPR1: poly-ADP-ribose polymerase 1; POLB: polymerase beta; POLG: polymerase gamma; POLD/E: polymerase delta/epsilon; LIG1/3: DNA ligase I/III; FEN1: flap endonuclease 1. (**b**) Nucleotide excision repair (NER) pathway, including transcription-coupled NER (TC-NER) and global genome NER (GG-NER) pathways. XPC/XPD: xeroderma pigmentosum complementation group C/D; RAD23B: UV excision repair protein radiation sensitive 23 homolog B; TFIIH: transcription initiation factor IIH; ERCC1/XPF: DNA excision repair protein 1/xeroderma pigmentosum complementation group F. (**c**) DNA mismatch repair (MMR) pathway. MutS/MutL: DNA mismatch repair proteins; EXO1: DNA exonuclease 1; PCNA: proliferating cell nuclear antigen. Figures were adapted from Ko et al. 2012 [85], Melis et al. 2013 [95] and Brierley et al. 2013 [101].

BER is the most prevalent process to repair oxidized DNA lesions both in nuclei and mitochondria, but using different protein components [72,79]. The classical cycle of BER is initiated by a series of DNA glycosylases that recognize and remove the non-bulky modified nucleotide bases. Among all the different DNA glycosylases present in the nuclei, only some of them have been detected in mitochondria, in which 8-oxoguanine DNA glycosylase 1 (OGG1) and uracil-DNA glycosylase (UNG) are the two main mitochondrial DNA glycosylases [80,81]. The DNA glycosylases cleave the *N*-glycosidic bond between the DNA lesion and deoxyribose, subsequently resulting in an apurinic/apyrimidinic (AP) site that recruits poly-ADP-ribose polymerase 1 (PARP1) and AP endonuclease 1 (APE1) [82,83]. Some bi-functional glycosylases also possess AP lyase activity that cuts the phosphodiester bond of DNA and creates a single-strand break [84]. APE1, located prominently in nucleus as well as in the mitochondrial matrix, has the capacity of creating termini specific for the newly to-be-inserted bases, which significantly activates PARP1 [85,86]. As the response to DNA damage, ADP-ribosylation by PARP1 triggers the recruitment of various components of the BER complex, including scaffold protein X-ray repair cross-complementing protein 1, bifunctional polynucleotide kinase, and gap-filling DNA polymerase beta (POLB) and DNA ligase III (LIG3) [87]. While POLB functions in nuclei, DNA polymerase gamma (POLG) functions only in mitochondria [88] (Figure 2a). By the time the BER complex is assembled, PARP1 accumulates enough negative charges for dissociation from the DNA lesion, enabling the BER complex to repair the damaged DNA [85]. 

Next to this classical short-patch BER, long-patch BER is activated in some particular cases, such as long-sequence oxidized DNA lesions (containing up to eight nucleotides) [89]. In this process, polymerase delta/epsilon (POLD/E), instead of POLB, are used to produce a nucleotide track, further becoming a single-strand DNA overhang. Additional enzymatic activity of flap endonuclease 1 is required in order to process such flap structure in long-patch BER both in nuclei and mitochondria [90]. Finally, this overhang inserts in the AP sites and then DNA ligase I (LIG1) in nuclei and LIG3 in mitochondria seal the nick [91] (Figure 2a). 

The upregulation of the BER pathway has been widely observed in various cardiovascular diseases. In an experimental heart failure model, myocardial DNA BER activity was upregulated through enhancing the capacity of elimination of oxidized products and enzymatic activities of relevant enzymes, including DNA glycosylase and APE1, playing an important role in counteracting the structural damage of tissue and myocardial remodeling during heart failure [92]. In addition, OGG1 was proven to repair 8-oxoG in human vascular smooth muscle cells, and thus to reverse the oxidative DNA lesions in atherosclerosis [93], the main cause of IHD [94].

### 3.2. Nucleotide Excision Repair (NER)

When oxidative stress results in bulky damage, or even whole DNA strand disruption, NER will take over. NER commonly consists of four steps: (1) DNA lesion recognition, (2) DNA helix unwinding, (3) incision making and subsequent excision of a damaged section and (4) DNA synthesis and ligation (Figure 2b). More than thirty proteins can be included in NER to handle a broad range of DNA damage types [95]. NER has two sub-processes: transcription-coupled NER (TC-NER) and global genome NER (GG-NER). The two sub-processes only differ in the initial recognition step. In GG-NER, the initial recognition of DNA lesions is achieved by the XPC-RAD23B complex, mainly comprised of xeroderma pigmentosum complementation group C and UV excision repair protein radiation sensitive 23 homolog B. In TC-NER, the function of this complex is replaced by a stalled RNA pol II complex, crucial for assembly of a number of TC-NER-associated enzymes [96]. Once the DNA lesion is recognized, the two sub-processes converge by the unwinding of DNA strands and exposure of bulky lesions using the base transcription initiation factor IIH and recruiting xeroderma pigmentosum complementation group D (XPD). Then, the NER machinery—DNA excision repair protein 1/xeroderma pigmentosum complementation group F (ERCC1/XPF)—cuts the damaged DNA sequences and new sequences will be regenerated by POLD and POLE. Finally, LIG1 seals the remaining nick [95].

The NER repair pathway is especially used for DNA lesions due to UV light and environmental mutagens [97], and several studies investigated the regulatory role of NER in the pathogenesis of cardiovascular disorders. In a study with *ERCC1* and *XPD* knockout mice, severe vascular dysfunctions such as enhanced vascular cellular senescence and abnormal vasodilator function were seen [98], indicating the potential implications of NER in pathophysiology of atherosclerosis. However, the direct role of NER in AF and IHD remains to be further explored.

### 3.3. DNA Mismatch Repair (MMR)

In addition to BER and NER, recent studies revealed that MMR also plays a crucial role in repairing oxidative DNA lesions. Until now, the molecular mechanism by which MMR eliminates DNA lesions had not been completely understood. It seems as if MMR-related proteins can distinguish between the parental strand and the newborn strand of DNA. Using the parental strand of DNA as the repair template, MMR corrects DNA lesions on the newborn strand [99,100].

There are two essential protein complexes in MMR: mammalian homologs of prokaryotic MutS and MutL [101]. MutS homolog proteins recognize and initiate the MMR process. After the recognition, MutL homolog proteins are recruited to the damaged site, enabling the DNA exonuclease 1 to cleave the damaged area, and next POLD/E, with the help of proliferating cell nuclear antigen (PCNA), generates new DNA sequences based on the parental template DNA strand. As a result, DNA is fully reconstituted, and LIG1 is assembled to patch the incision (Figure 2c).

Currently, there is still a limited amount of research correlating the MMR pathway and heart diseases. Among these, one study revealed a possible regulation of MMR in human heart failure. The diminished human MutY homolog, a BER pathway related DNA glycosylase proven to functionally interact with human MutSα to promote MMR repair process [102], was remarkably associated with the elevated level of 8-oxoG, implying a possible novel therapeutic direction for heart failure treatments [103]. 

Collectively, three different DNA repair pathways protect the DNA molecule from oxidative damage. Accumulating evidence shows that the deficient capacity of BER, NER, and MMR was remarkably corelated with several cancers and other disorders, including cardiac diseases [50,95,103,104]. While DNA repair is well studied in some diseases, it is inadequately clarified in others. It is worthwhile to explore the role of DNA repair pathways in these diseases, as they may provide novel therapeutic targets for oxidative DNA damage-induced disorders, especially AF and IHD.

## 4. Oxidative DNA Damage and Repair in AF

In general, the pathophysiology of AF consists of roughly two stages: the initiation of the arrhythmia and the maintenance and progression of the disease to longer lasting forms. AF induction requires vulnerable substrates as well as triggers to initiate pathophysiological alteration [105]. Various clinical conditions, such as heart failure, hypertension, metabolic syndrome, aging and diabetes, induce AF promoting factors, which create substrates and/or triggers responsible for the first-onset of AF (primary stage) [106,107,108,109,110]. Once AF is initiated, atrial arrhythmogenic remodeling, characterized by reversible electrical remodeling and sustainable structural remodeling of cardiomyocytes, is central for the maintenance and progression of AF (secondary stage) [111,112]. When AF persists, there is a significant increase of the likelihood of developing a wide range of potential complications, such as stroke, heart failure and myocardial infarction, which could contribute significantly to population mortality [3,113,114,115].

Oxidative stress as one of key promoting factors of AF can cause Ca^2+^ overload, as well as atrial fibrosis, myolysis, and hypertrophy, which are associated with atrial electrical and structural remodeling and contribute to the onset and progression of AF [116,117]. In AF patients, an increase in oxidative DNA damage markers (i.e., 8-oxoG and 53BP1) [26] and differentially expressed proteins, closely related to oxidative stress-related signaling pathways [116], have been observed. Moreover, serum levels of 8-hydroxy-2′-deoxyguanosine (8-OHdG), a vital oxidative DNA damage by-product formed by exonucleases during DNA repair processes, positively correlate with AF progression, onset of postoperative AF (poAF), and recurrence after AF treatment [118], substantiating a role for oxidative stress-induced DNA damage in AF development. In the next part of this review, two major underlying biochemical events associated with oxidative DNA damage and repair in AF and a summary of novel therapeutic treatment implications for AF will be discussed. 

### 4.1. Oxidative Mitochondrial DNA Damage and Dysfunction in AF

Since atrial contraction and cellular function of cardiac tissue rely heavily on mitochondria for oxidative energy generation, and mitochondrial ROS-induced oxidative stress is associated with AF and its promoting conditions, mtDNA damage could play a central role in the pathophysiology of AF. Several studies explored the relationship between oxidative mtDNA damage/deletion) and the onset of AF. Increased oxidative injury and deletion of mtDNA were observed in atrial muscle of patients with AF, which might contribute to dysfunctional mitochondria that underlie the disease progression of AF [30]. AF occurrence increases with old age, resulting in metabolic dysfunction associated with the mtDNA deletion, causing a pro-arrhythmic substrate [119,120]. Tsuboi et al. discovered 7.4 kb of mtDNA deletion with decreased level of adenine nucleotides in atrium tissues of elderly AF patients, suggesting its association with mitochondrial dysfunction by impairing ATP synthesis [121]. Further research using a larger sample size of AF patients found that AF was associated with an accumulation of aging-related common type mtDNA deletion mutation in human atrial tissue [122].

In addition to the direct evidence for the occurrence of mtDNA lesions, mitochondrial dysfunction was widely found in AF in various studies (Table 1). All these studies showed that mitochondrial function was compromised in patients and in animal models with AF [123,124,125,126]. For instance, cardiopulmonary bypass-induced inflammation and oxidative stress could trigger the mitochondrial dysfunction by altering major pathways for cellular and mitochondrial energy supply. This malfunction predisposes the onset of AF [125]. Evidence also suggested that conservation of mitochondrial function protects against tachypacing-induced cardiomyocyte remodeling in AF models [126]. Mitochondrial dysfunction affects cardiomyocyte metabolism with respect to structural, contractile, and electrophysiological properties in AF, and thus understanding mechanisms by which mtDNA damage, as a cause for mitochondrial dysfunction, contributes to the development of AF can potentially offer substantial therapeutic benefits for AF patients.

### 4.2. The Oxidative Nuclear DNA Damage—Excessive PARP1 Activation—NAD^+^ Depletion Axis in AF

Interestingly, a recent study revealed a significant and consistent increase of nuclear and mitochondrial DNA damage in experimental and human AF, where the recruitment of nuclear DNA repair machineries was occurring by activation of PARP1, a major NAD^+^ consumer [26]. This newly elucidated responding axis in AF, involving oxidative nuclear DNA damage, excessive PARP1 activation and NAD^+^ depletion, opens a new avenue to better understand the pathophysiological mechanisms underlying AF.

PARP1 is the most abundant cellular ADP-ribosyl transferase and uses NAD^+^ as substrate. PARP1 senses DNA single-strand breaks generated directly or serves to recruit the BER DNA lesion repair machinery [127]. PARP1 has three different functional domains: the DNA-binding domain (zinc fingers), the auto-modification domain, and the catalytic domain. Normally, when mild DNA damage occurs, zinc fingers can efficiently recognize and bind to the DNA single/double strand break sites, followed by poly-ADP-ribose (PAR) formation promoted through the auto-modification domain. After that, the catalytic domain is stimulated dramatically, up to 500-fold, and is responsible for building up the structure of PAR by transferring ADP-ribose subunits using NAD^+^ as substrate [128,129]. Because of the increasing electrons density conferred by the extending PAR polymer, DNA repair-related proteins, such as the BER complex, are recruited [130]. However, growth of PAR polymer is controlled by the PAR glycohydrolase or ADP-ribosyl-acceptor hydrolase 3, which cleaves PAR from PARP1 complex [131]. Then, PARP1 is released for recognition of the next DNA lesion and initiation of recruitment of DNA repair proteins [132]. 

**Figure 3:**
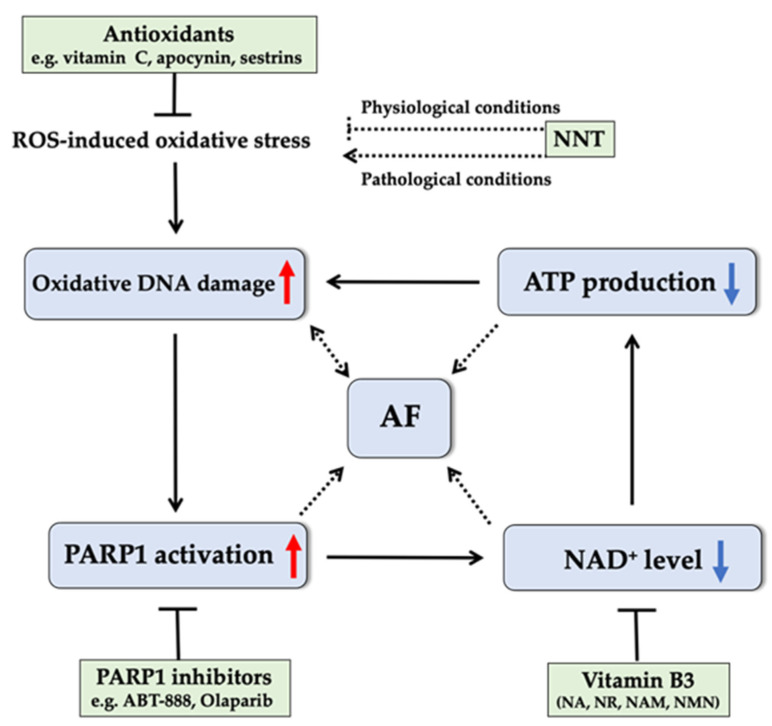
Schematic representation of the vicious cycle of “oxidative DNA damage-excessive PARP1 activation and NAD^+^ depletion” in the pathophysiology of AF and potential therapeutic role of antioxidants, PARP1 inhibitors, and vitamin B3 in AF progression. Oxidative DNA damage caused by oxidative stress activates PARP1, initiating the depletion of NAD^+^, a key coenzyme associated with redox balance and energy metabolism. Subsequent failure to meet the increased energy demand during AF episodes further exacerbates oxidative DNA damage, and electrical and contractile dysfunction in AF, initiating a vicious cycle. Antioxidants, PARP1 inhibitors, and NAD^+^ replenishment by various dietary forms of vitamin B3 could preclude this vicious cycle, implicating their novel potential therapeutic role in oxidative DNA damage-induced AF. As a key antioxidative enzyme, NNT plays an important role in mitochondrial redox homeostasis under normal physiological conditions. Its mechanistic role in pathological conditions in the heart remains to be investigated. PARP1: poly-ADP-ribose polymerase 1; AF: atrial fibrillation; NAD^+^: nicotinamide adenine dinucleotide; NNT: nicotinamide nucleotide transhydrogenase; NA: nicotinic acid; NR: nicotinamide riboside; NAM: nicotinamide; NMN: nicotinamide mononucleotide.

Compared with mild stress, intermediate and severe oxidative stress conditions in AF induce more DNA damage and lead to excessive PARP1 activation, which in turn consumes NAD^+^ to such an extent that cellular NAD^+^ is depleted [26,133]. NAD^+^ also functions as an essential cofactor in mitochondrial redox reactions and energy metabolism and is of particular relevance to cells with a high metabolic activity, such as cardiomyocytes [134,135]. Therefore, depleted NAD^+^ levels by overactivation of PARP1 induces a scenario of oxidative stress and energy deficit. Subsequent failure to meet the increased energy demand due to elevated electrical and contractile activity during AF episodes further exacerbates progressive mitochondrial dysfunction, oxidative DNA damage, and electrical and contractile dysfunction, initiating a vicious cycle (Figure 3). NAD^+^ has been shown to play a unique role in DNA repair mechanisms [134], and recent studies have shown that administering PARP inhibitors or NAD^+^ replenishment drugs could preclude this vicious circle by attenuating oxidative DNA damage and counteracting structural remodeling, electropathology, and contractile dysfunction in atrial cardiomyocytes [26], implicating their novel therapeutic role in oxidative DNA damage-induced AF. 

Furthermore, mitochondrial NAD^+^ can be partly produced by mitochondrial enzyme nicotinamide nucleotide transhydrogenase (NNT) from its reduced form (i.e., NADH). NNT uses NADH as an electron donor to generate NADPH under physiological conditions [136], and thus has a key role in maintaining redox homeostasis. In view of the high levels of NNT especially in heart tissue [137] and the relation between the inactivating mutations in *NNT* gene and cardiovascular disorders [137,138], it would be worthwhile to study the role of NNT as a modulator for AF. This is especially relevant because it has been reported that a *NNT* mutation impaired mitochondrial function and energy metabolism, which likely increased AF incidence, also due to a disturbed cellular redox imbalance and possibly to reduced NAD^+^ availability [26,123,124,136,139]. Furthermore, in pathological conditions, reversal of NNT could occur to support ATP production by depleting NADPH, leading to increased oxidative stress [140] (Figure 3). Therefore, the exact role of NNT in regulating oxidative stress and AF progression needs to be carefully examined. 

### 4.3. Novel Therapeutic Strategies for AF

The prevalence of AF is expected to double in the next decades, becoming a global medical challenge [141,142,143]. Currently, most of the commonly used AF treatments are focused on surgical and anti-arrhythmic pharmacotherapeutic treatments [14]. Although invasive catheter-based ablation is promising in early-stage AF, a high percentage of recurrence requires even multiple expensive procedures [144]; currently available pharmacological therapies such as amiodarone, digoxin, calcium-channel blockers, and beta-blockers are mostly symptomatic treatments, directed at rate/rhythm control [4,145]. They do not prevent AF progression from paroxysmal to final permanent AF and have potentially severe (fatal) side effects. The difficulty in treating AF predicts a great need for dissecting the root causes of AF with an ultimate goal of developing therapies focused on its core pathological mechanisms. Interestingly, new findings reveal oxidative DNA damage as a mechanistic root cause of AF [26]. Hence, nutritional/nutraceutical and pharmacological interventions that target pathways of oxidative DNA damage and repair represent novel therapeutic options for AF.

#### 4.3.1. Antioxidants: Reducing Upstream Oxidative Stress

To reduce oxidative DNA damage, antioxidants represent a potentially effective treatment for AF by reducing upstream oxidative stress. In basic experimental studies and clinical trials, disturbance of the ROS detoxification system has been implicated in atrial structural remodeling damage, contributing to the pathogenesis of AF [146]. Hence, it is conceivable that inhibition of cardiac ROS-mediated oxidative stress might be used for developing effective therapies to successfully manage the onset of AF [147].

Antioxidant treatments were shown to have some preventive effects against the induction and development of (po)AF [148,149,150,151]. Vitamin C (VitC) treatment is one of the most commonly used antioxidant therapies, especially in the medical field of poAF. In a meta-analysis that included a total of 2050 high-risk AF patients from different countries, administration of VitC on average lowered the incidence of poAF by more than 25% [152]. Interestingly, cases in developing countries showed a much higher efficacy compared with those in well-developed countries, possibly due to differences in nutritional status or hospital management [153,154,155]. Another study, however, found an unremarkable benefit of long term oral VitC administration on prevention of AF, especially for middle-aged and older people [156], implying that an additional dose of VitC, beyond daily requirement, does not provide any additive effect on AF prevention.

Another antioxidant strategy is to target relevant proteins prior to ROS production [157]. Targeting the signals belonging to the upstream ROS-induced oxidative stress pathway would also be a possible therapeutic treatment for AF. Apocynin, as an antioxidant, could pre-emptively prevent ROS formation and markedly block the induction and duration of AF by regulating the expression of oxidative stress-related proteins and by inhibiting the increased Ca^2+^ release in sarcoplasmic reticulum to attenuate atrial electrical remodeling [116]. In addition, sestrins, which are stress-related proteins, accumulate when cells are exposed to detrimental environments, such as hypoxia, oxidative stress and DNA damage [158]. The upregulation of sestrins against ROS accumulation and Ca^2+^ overload [159], was revealed for the first time by Dong et al. to protect atria against oxidative damage and fibrosis in both experimental and clinical AF [160]. Although the physiological mechanisms of sestrin function still remain poorly understood, this type of antioxidant may have potential as an endogenous protective target in clinical management of AF.

#### 4.3.2. PARP1 Inhibitors and NAD^+^ Supplements: Targeting the Pathway of Oxidative DNA Damage and Repair

Given the detrimental effects of oxidative stress-induced DNA damage on AF occurrence and maintenance, antioxidative DNA damage therapy can be expected to be at the center of AF treatment. An increasing number of studies revealed regulatory roles for factors associated with oxidative DNA damage and repair pathways in the reversal of many cardiac diseases, making them potential therapeutic targets in heart diseases. As mentioned above, a recent study showed that AF was associated with excessive PARP1 activation precipitated by oxidative DNA damage [26]. Activated PARP1 in turn consumed NAD^+^, resulting in contractile dysfunction in tachypaced cardiomyocytes and persistent AF patients. This uncovered PARP1 as a potential oxidative DNA damage and repair mechanism-based therapeutic target for preventing AF. It was revealed that pharmacological PARP1 inhibitors, including ABT-888, Olaparib and nicotinamide (NAM), counteracted NAD^+^ depletion, precluded oxidative DNA damage, and further preserved atrial contractile dysfunction in experimental AF (Figure 3). Future research should elucidate the translational potential of therapeutic targeting of the oxidative DNA damage-induced PARP1 activation pathway in clinical AF.

Another promising therapeutic option to prevent the vicious cycle of “oxidative DNA damage—excessive PARP1 activation and NAD^+^ depletion” in AF is through exogenous replenishment of NAD^+^ and/or its various forms of precursors [26]. NAD^+^ can be synthesized *de novo* from tryptophan but is mostly acquired via various dietary forms of vitamin B3 (VitB3): nicotinic acid (NA), NAM, nicotinamide riboside (NR), and nicotinamide mononucleotide (NMN) [161,162]. In particular, supplementation of NA, NMN, or NR has been shown as an effective strategy for boosting NAD^+^ levels in mouse models of aging [163,164,165], vascular diseases [166], and diabetes [167]. High dose NA supplementation can cause side effects, such as flushing [168]. While high dose NR supplementation has shown effectiveness in some disease models (e.g., obesity [169], diabetic neuropathy [170], dilated cardiomyopathy [171]), adverse effects on metabolism were also observed in overweight but otherwise relative heathy conditions, which might be attributed to the specific *NNT* containing genotype of mouse model [172,173]. This could suggest that efficacy of VitB3 supplementation is dependent on the presence of health conditions with a high NAD^+^ demand, such as those occurring in AF. VitB3, like other B-vitamins, has a key role in mitochondrial metabolism [174]. Sufficient VitB3 intake is essential to maintain mitochondrial function, balance levels of mitochondrial signaling metabolites, and prevent metabolic stress, while a dysregulated VitB3 metabolism leads to increased levels of mitochondrial ROS emission and oxidative DNA damage [175]. VitB3 thus may offer substantial therapeutic benefits for AF initiation and progression (Figure 3). Various dietary forms of VitB3 were shown to slow down oxidative stress-related heart diseases mostly by affecting NAD^+^ biosynthesis. Of potential relevance, NAM is not only a NAD^+^ precursor, but also a PARPs inhibitor [176], potentially exerting a dual pharmacodynamic effect on cardiomyocyte remodeling in AF. Interestingly, it was reported that NAM supplementation prevented experimental AF [177], also showed an amelioration of left ventricular contractile dysfunction, and attenuated progression of cardiac hypertrophy and heart failure by normalizing NAD^+^ levels [178]. NR represents a potential therapy for diseases in which NAD^+^ depletion has been implicated, such as AF and heart failure [179]. Supplementation of NMN, but also with NAM or with NR, has the potential to attenuate NAD^+^ imbalance and oxidative DNA damage as promoting factors of AF induction and maintenance. 

In conclusion, both pharmacological inhibitors of PARP1 and nutritional supplements of NAD^+^ (in the form of VitB3) can break the vicious circle caused by oxidative DNA damage in AF and thus represent novel therapeutic options for AF.

## 5. Oxidative DNA Damage and Repair in IHD

### 5.1. Oxidative DNA Damage and IRI in IHD

**Figure 4:**
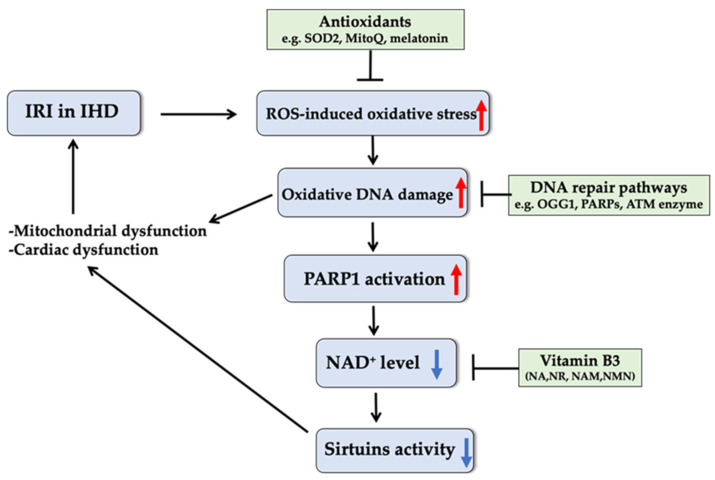
Schematic relation between oxidative stress-induced DNA damage and the pathophysiology of IRI in IHD and potential therapeutic role of antioxidants, vitamin B3, and enzymes involved in DNA repair pathways in IRI treatment. Series of pathophysiological modifications during reperfusion injury stimulate net ROS emission, which induces oxidative DNA damage and activates PARP1 enzyme, resulting in depletion of intracellular NAD^+^. Since sirtuins depend on intracellular NAD^+^ for their deacetylase activity, depleted NAD^+^ leads to reduction in activity of sirtuins, which impairs mitochondrial functions and antioxidant defense, leading to further aggravation of IRI. Antioxidants and enhancement of oxidative stress defense and DNA repair pathways as well as NAD^+^ replenishment by various dietary forms of vitamin B3 could provide protection against IRI in IHD through reduction of oxidative stress and DNA damage and promotion of mitochondrial function and DNA repair capacity. IHD: ischemic heart disease; IRI: ischemia/reperfusion injury; PARP1: poly-ADP-ribose polymerase 1; ATM: ataxia telangiectasis mutated; NAD^+^: nicotinamide adenine dinucleotide; NA: nicotinic acid; NR: nicotinamide riboside; NAM: nicotinamide; NMN: nicotinamide mononucleotide; SOD2: superoxide dismutase 2; OGG1: 8-oxoguanine DNA glycosylase 1.

IHD is one of the leading causes of mortality in the world [180]. IRI is the most common consequence of IHD, generally resulting in necrosis and apoptosis, as well as a transient reduction of contractility of surviving myocardium [181]. In ischemic conditions, anaerobic glycolysis plays the dominant role in producing ATP within heart muscles after a very short time of complete or partial obstruction of coronary arteries, and excess H^+^ is intracellularly synthesized during glycolysis [181,182]. Once reperfusion and reoxygenation occur, when blood re-enters the tissue following ischemia, the cardiac tissue can only work at a quite ineffective rate due to aggravated arrhythmias, microvascular injury, and myocardial dysfunction, etc. [183,184,185], mainly resulting from the imbalance of electrolytes [186]. Reperfusion results in a ROS burst, which is a major contribution to reperfusion injury. The ROS burst and the series of pathophysiological modifications in the course of IRI resulting from an extreme mitochondrial redox condition further stimulate net ROS emission. Excessive ROS production induces oxidative DNA damage and activates PARP1, resulting in depletion of intracellular NAD^+^ and ATP [187,188]. NAD^+^ is also a rate-limiting co-substrate for sirtuin family proteins (SIRT1-7), which all serve as important regulators of redox homeostasis and are implicated in various cardiac diseases [189,190]. NAD^+^ depletion will thus lead to reduction in the activity of SIRT1 [191] and SIRT3 [192], which impairs mitochondrial biogenesis and antioxidant defense, further enhancing mitochondrial dysfunction, one of the hallmarks of IRI [193] (Figure 4). 

Since 1998, numerous studies revealed and validated the link between myocardial IRI and ROS-induced oxidative DNA damage. In an *ex vivo* global ischemia mouse model, mtDNA damage was accumulated in the post-ischemia condition [194]. A clinical study showed that human ischemic hearts have increased mtDNA damage and oxidative phosphorylation deficiency [76]. A study utilizing isolated rat hearts proved that the synthesis of 8-oxoG, a biomarker for oxidative DNA damage, was positively correlated with the severity of IRI [195]. Similarly, the level of 8-OHdG was steadily increased as a function of reperfusion time in rat myocardium and was completely blocked when hearts were given a ROS scavenger [196], suggesting a considerable role for oxidative DNA damage in the pathogenesis of myocardial IRI. Moreover, malfunctions of DNA repair proteins were reported to cause defects in cell proliferation, apoptosis, and mitochondrial dysfunction, which in turn increase the incidence of metabolic syndromes, atherosclerosis, and IHD [197,198]. However, although it is essential for interventional strategies to protect the heart from ischemic injury, such strategies are inevitably complicated by reperfusion injury. Insufficient clinical trial data and lack of pharmacokinetic and pharmacodynamic studies prompt critical demands for effective therapies for IRI in IHD.

### 5.2. Potential Therapies for IRI Recovery in IHD

IRI-induced DNA damage can not only directly cause the cardiac dysfunction but also in turn aggravate the development of IRI [199]. Therefore, therapeutic targets at oxidative stress and oxidative DNA damage and repair pathways are expected to offer benefits for recovery from IRI in IHD.

#### 5.2.1. Antioxidants: Attenuating the Oxidative DNA Damage in IRI

To overcome the oxidative stress, animals have a complex antioxidant defense system to prevent damage to DNA (Figure 4). Highly reactive superoxide is converted to hydrogen peroxide, and thus is largely inactivated by SOD2, an antioxidant enzyme that resides inside mitochondria. Overexpression of SOD2 was reported to decrease the level of ROS-related DNA damage and effectively limit the size of murine myocardial infarct [200,201,202]. Additionally, in a study using a murine heart transplantation model, the presence of a mitochondrial-specific antioxidant, MitoQ, was demonstrated to lower oxidative DNA damage and reduce the early stage of pro-inflammation in the recipient rat [203]. This finding offers the potential to improve the supplement of heart grafts and suppress the associated cardiac injury after the transplantation. The third antioxidant tested for IRI treatment is melatonin. As an antioxidant, melatonin can directly carry out its oxidation-resisted capacity via protecting Tom70, a mitochondrial translocase, which is considered as a repressor of oxidative stress [204]. On the other hand, melatonin is also involved in the c-Jun N-terminal kinase pathway, downregulating c-Jun N-terminal kinase expression, thus lowering the stimulation of oxidative stress [205]. Collectively, these antioxidants will enable potentially therapeutic treatments for IRI and related complications by targeting oxidative stress-induced DNA damage, while precise underlying mechanisms of action and the translation of antioxidants to therapeutic use remain in need of further exploration. 

#### 5.2.2. Novel NAD^+^-Based Therapeutic Approaches for IRI Recovery

Ischemia followed by reperfusion can be counteracted by a mechanism known as ischemic preconditioning [186]. Ischemic preconditioning induces activation of SIRT1 [206], which causes deacetylation of FoxO3 transcription factor that is responsible for ROS generation [207], and thus prevents injury due to IRI. SIRT1 depends on intracellular NAD^+^ for its deacetylase activity [208] and is activated by higher NAD^+^ levels, thus mimicking the action of ischemic preconditioning to ameliorate IRI. Yamamoto et al. revealed that the level of NAD^+^ is significantly reduced during ischemic states [209]. Therefore, SIRT1 activation by restoring the cellular NAD^+^ levels through supplementation with exogenous NAD^+^ or its precursors (i.e., dietary forms of VitB3) may aid in the restoration of mitochondrial redox homeostasis and combat oxidative DNA damage and IRI progression (Figure 4).

Indeed, NAD^+^ supplementation was shown to alleviate IRI damage via SIRT5 [210]; downregulation of SIRT2 was involved in protection against IRI [211], and a protective role of SIRT4 against IRI was found to be associated with preserved mitochondrial function and decreased myocardial apoptosis [212], indicating direct therapeutic roles for SIRT proteins in IRI treatments. In addition, a number of studies have demonstrated that increasing cellular NAD^+^ levels protected against IRI in cardiac tissue through reducing oxidative stress and promoting mitochondrial function and antioxidant capacity. Zhang et al. found that intravenous administration of NAD^+^ significantly attenuated rat myocardial IRI by enhancing the cardiac antioxidant capacity [213], while Zhai et al. reported that exogenous supplementation of NAD^+^ protected swine myocardium from IRI characterized through less cardiac fibrosis and better ventricular compliance [214]. Moreover, preclinical studies have demonstrated pharmacological activities of dietary forms of VitB3 in cardiac ischemia where a NAD^+^ deficit had been indicated. Specifically, NR was shown to alleviate the myocardial IRI by improving mitochondrial biogenesis [215]. In addition to induction of activation of SIRT1 [163,208], a study revealed that NMN offered an acute cardioprotective function against IRI partly by direct stimulation of glycolysis or acidification [216]. When NMN is provided during ischemia, glycolysis is increased to facilitate ATP production, thus promoting cardioprotection, while if NMN is given during reperfusion, it protects the heart by enhancing acidosis, which is known to be cardioprotective during early reperfusion via a shutdown of mitochondrial permeability transition pore to maintain the mitochondrial membrane potential and ATP balance [217,218].

#### 5.2.3. Therapeutic Enzymes Involved in Oxidative DNA Damage and Repair Pathways

The repair of oxidized DNA lesions can be a direct therapeutic target of IRI and associated complications in IHD. Several key enzymes directly involved in oxidative DNA repair have already drawn substantial research attention (Figure 4). Among these, OGG1, as part of BER, removes oxidative DNA lesions, mainly 8-oxoG, and maintains the DNA integrity under oxidative stress [194]. Using post-ischemic rat hearts, it was shown that an enhanced level of 8-oxoG caused an increase in OGG1 [96]. Overexpressing OGG1 was shown to decrease mtDNA damage and reduce mouse myocardial fibrosis following aortic banding [219]. In addition, synergism between OGG1 and the DNA glycosylase homologous to MutY was also responsible for preservation of mtDNA in an *ex vivo* IR model [194]. OGG1 therefore provides a promising target in the prevention of IRI in IHD. In addition, the DNA repair enzymes PARPs were proven to modulate pathophysiology of myocardial injury caused by myocardial infarction and IRI [220,221,222]. Activation of PARPs in a rat myocardial ischemia–reperfusion model has been detected [223], and inhibition of PARPs by 3-aminobenzamide or 1,5 didroxyisoquinoline reduced infarct size and restored myocardial contractility caused by ischemia and reperfusion in the rabbit and rat *in vivo* [220,223,224], suggesting that pharmacological inhibition of PARPs is a viable approach for protection against myocardial IRI and related cardiac disorders. Furthermore, the phosphoinositol 3-kinase-like serine/threonine protein kinases ataxia telangiectasis mutated (ATM), as a sensor to DNA damage by phosphorylating key substrates involved in DNA repair pathways, played a cytoprotective role against myocardial [225] and renal IRI [226], providing a potential therapeutic target for recovery from IRI. However, it would be worthwhile to further investigate the underlying mechanisms of the effects of OGG1, PARPs, and ATM on IRI, as well as the role of other components of DNA repair pathways in IHD.

In conclusion, some antioxidants, replenishment of NAD^+^/VitB3, and enzymes directly involved in DNA repair pathways provide novel therapeutic options to prevent oxidative DNA damage-induced IRI in IHD. However, for development of effective and sustainable therapies, interference with pathophysiological and pharmacological mechanisms of action are mechanistically incomplete and need further investigation.

## 6. Summary

The morbidity and mortality of AF and IHD are expected to rise sharply, becoming a global medical challenge. The limited understanding of cellular and molecular mechanisms behind oxidative DNA damage-related pathophysiology impedes the development of effective target-specific therapeutic strategies for AF and IHD. In this review, we focused on the potential role for oxidative DNA damage and repair in AF and IHD and discussed a vicious cycle in which excessive ROS-induced oxidative DNA damage results in depletion of intracellular NAD^+^ and ATP, shifting the redox environment to a state of energy deficit and a compromised mitochondrial ROS scavenging capacity, further exacerbating DNA damage and myocardial dysfunction in AF and IHD. Therefore, mitochondrial function, DNA repair, antioxidant defense, and energy and NAD^+^ homeostasis all represent major targets for future AF and IHD treatment. Detailed and systematic investigations of mechanisms underlying the interaction between oxidative DNA damage/repair and the pathology of AF and IHD are still limited and are urgently needed. Performing clinical studies with NAD^+^ (VitB3) replenishment and drugs directed at oxidative DNA repair pathways deserve strong priority in order to validate their potential for prevention and treatment of AF and IHD. 

## Figures and Tables

**Table 1:** Summary of the main studies investigating the relationship between mitochondrial dysfunction and pathophysiology of atrial fibrillation.

Literature ^1^	Subjects	Measurements	Results
Montaigne et al. [123]	- right atrial muscles from 104 potential poAF patients undergoing coronary artery bypass graft surgery	- pre-operative mitochondrial respiration - Ca^2+^ retention capacity - respiratory complex activity	- lower mitochondrial respiratory rate - decreased Ca^2+^ retention capacity - inhibited respiratory complex activity
Xie et al. [124]	- atrial myocytes from 10 patients with chronic AF - RyR2-S2808D^+/+^ mice model with constitutively leaky RyR2 channel	- oxidation of RyR2 - mitochondrial ROS level	*AF patients’ samples:* - RyR2 oxidation, leading to Ca^2+^ channel leak and dysfunctional cellular electric activity *RyR2-S2808D^+/+^ mice models:* - increased AF susceptibility - increased intracellular oxidative stress - greater mitochondrial ROS production
Jeganathan et al. [125]	- right atrial tissue and serum samples from 85 potential poAF patients before and after cardiopulmonary bypass	- genetic and proteic biomarkers for oxidative stress and mitochondrial damage: DHCR24, CREB, pBcl-2/Bcl-2, cleaved Caspase-9, CIDEC	- DHCR24, meaning higher oxidative stress - decreased CIDEC and pBcl-2/Bcl-2, increased CREB, and cleaved Caspase-9, meaning higher mitochondrial damage
Wiersma et al. [126]	- tachypaced HL-1 atrial cardiomyocytes - atrial appendages from AF patients in different stages	- CaT_mito_ amplitudes, for mitochondrial Ca^2+^-handling - ATP level - ROS level - mitochondrial morphology and membrane potential - OCR - HSP10 and HSP60, mitochondrial stress-related chaperones	- lower mitochondrial Ca^2+^-handling - lower mitochondrial membrane potential - lower ATP production - lower respiratory rate - fragmented tubular network of mitochondria - higher level of HSP10 and HSP60

^1^ Publications are listed in the order of published time. poAF: post-operative atrial fibrillation; RyR2: type 2 ryanodine receptor; CIDEC: cell death inducing DNA fragmentation factor subunit alpha like effector C; CREB: cyclic adenosine monophosphate-responsive element binding protein; DHCR24: 24-dehydrocholesterol reductase; CaT_mito_: mitochondrial calcium transient; OCR: oxygen consumption rate; pBcl-2/Bcl-2: phosphorylated B-cell lymphoma 2/B-cell lymphoma 2; HSP: heat shock protein.

## Data Availability

No original data were created or analyzed in this study.
